# New insights into the immunomodulatory properties of poxvirus cytokine decoy receptors at the cell surface

**DOI:** 10.12688/f1000research.14238.1

**Published:** 2018-06-11

**Authors:** Bruno Hernaez, Antonio Alcami

**Affiliations:** 1 Centro de Biología Molecular Severo Ochoa (Consejo Superior de Investigaciones Científicas and Universidad Autónoma de Madrid), Nicolás Cabrera 1, Cantoblanco, 28049 , Madrid, Spain

**Keywords:** Poxvirus, Immune evasion, cytokine receptor, interferon, chemokine, glycosaminoglycan

## Abstract

Poxviruses encode a set of secreted proteins that bind cytokines and chemokines as a strategy to modulate host defense mechanisms. These viral proteins mimic the activity of host cytokine decoy receptors but have unique properties that may enhance their activity. Here, we describe the ability of poxvirus cytokine receptors to attach to the cell surface after secretion from infected cells, and we discuss the advantages that this property may confer to these viral immunomodulatory proteins.

## Introduction

Poxviruses are well-known host immune evaders. To counteract the different components of the innate and adaptive immune responses, they have evolved diverse and often unique mechanisms of viral evasion. Poxviruses encode a large plethora of genes encoding immunomodulatory proteins, including secreted homologues of host cytokines, chemokines, and their receptors, directed to subvert the antiviral effect of host immune responses ^1– 3^. Most of the virus-encoded cytokine decoy receptors are secreted from infected cells into the medium and exert their inhibitory function as a soluble form ^4^. Only a few of them have been additionally described to attach to cell surfaces after secretion from infected cells and to function from this location. In this review, we focus on these immunomodulatory proteins encoded by poxviruses: the interleukin-18-binding protein (vIL18-BP), the type I interferon-binding protein (vIFNα/βBP), the chemokine-binding proteins (vCKBPs) A41 and M-T1, and the complement control protein.

These virally encoded proteins share the ability to directly bind glycosaminoglycans (GAGs) with high affinity to attach to the cell surface during infection. GAGs are complex linear, often sulfated, anionic polysaccharides, usually bound to a core protein to constitute the proteoglycans found as components of the extracellular matrix or inserted into the plasma membrane of virtually all mammalian cells. Protein interactions with GAGs often occur through basic amino acid clusters, including lysine, arginine, or histidine residues, which form the canonical GAG-binding motifs BBXB and BBBXXB, where B is a basic residue and X is any amino acid. The different composition of the GAGs defines the binding sites not only for virus proteins but also for a multitude of specific ligands, including cytokines, chemokines, growth factors, enzymes, and enzyme inhibitors. Thus, a variety of cell functions have been ascribed to GAGs, such as cell attachment, regulation of cellular proliferation, cell migration, morphogenesis, tissue repair, or viral pathogenicity ^5, 6^.

The interaction with cell surfaces may confer to these secreted poxvirus proteins the same advantages as those observed for other mammalian GAG-binding proteins: (i) a retention mechanism to act in the vicinity of infection sites, where their regulatory function is most required; (ii) a local increase in their concentration, enhancing their function and avoiding clearance by the surrounding plasma/media flow; (iii) a longer persistence attached to the cell surface, escaping degradation from plasma proteases; and (iv) a location to interact with other molecules. In this review, we summarize the specific functional aspects of each of the above-mentioned poxvirus immunomodulators with special emphasis on the impact of the GAG-binding properties on their inhibitory function.

## vIL-18BP

IL-18 is a proinflammatory cytokine and a member of the IL-1 cytokine superfamily and promotes a Th1 host response through the potent induction of IFN-γ and natural killer cell activity ^7^. Indeed, it was originally named “IFN-γ-inducing factor” after its first description. It was soon demonstrated that IL-18 played an important role during poxvirus infections since exogenous IL-18 protected mice from vaccinia virus (VACV) infection ^8^. Furthermore, simultaneously to the description of the soluble IL-18-binding proteins (IL-18BPs) from mice and humans ^9^, a homologue of viral origin (vIL-18BP) was found in molluscum contagiosum virus (MCV), a human poxvirus that causes benign skin lesions that persist for months without signs of inflammation ^10, 11^. Since then, vIL18-BP homologues have been found in many other members of the orthopoxvirus genus, such as VACV, cowpox virus (CPXV), and ectromelia virus (ECTV) ^12– 14^.

**Figure 1.**
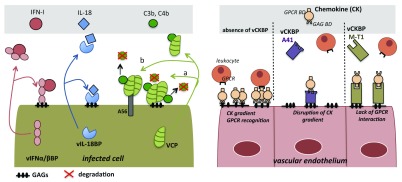
Poxvirus secreted immunomodulators function at the cell surface. Viral IL-18-binding protein (vIL-18BP) and viral IFN-I-binding protein (vIFNα/βBP) are secreted from infected cells and bind their respective ligands either as a soluble form or anchored to the cell surface through glycosaminoglycan (GAG) interactions, acting in both cases as cytokine decoy receptors. Vaccinia virus complement control protein (VCP) retention at the cell surface is mediated by either GAGs (a) or the viral protein A56 (b) and binds regulatory complement proteins promoting their inactivation. A41 and M-T1 viral chemokine-binding proteins (vCKBPs) are anchored to the surface of endothelium by interacting with GAGs and simultaneously bind chemokines. A41 interacts with the chemokine GAG-binding domain (GAG BD) to disrupt the chemotactic gradient, while M-T1 interacts with the G-protein-coupled receptor (GPCR)-binding domain (GPCR BD) in the chemokine to avoid leukocyte recognition. IFN-I, interferon type I; IL-18; interleukin-18.

vIL18-BPs lack similarity to the cellular membrane IL-18 receptor but are structurally related to human IL-18BP, which is also secreted from cells and can neutralize IL-18-induced effects by directly binding to this cytokine with high affinity, acting as a decoy receptor ^13, 15, 16^ ( Figure 1).

Structural studies have identified two aromatic residues on vIL-18BPs that are critical for their interaction with IL-18 and conserved among poxviruses ^17^ with the exception of the more distantly related yatapoxvirus genus ^18^. In addition to IL-18 binding, vIL-18BPs from variola virus (VARV) and MCV exhibit GAG-binding properties to attach to the cell surface ^11, 19^. In this case, the clusters of positively charged residues required for GAG binding were identified at the carboxy-terminal region of vIL-18BPs. Interestingly, the carboxy-terminal region of the vIL-18BPs from VACV, monkeypox virus (MPXV), and some ECTV strains, including Naval, is identical to the vIL-18BP from VARV and believed to bind to the cell surface. However, the vIL-18BP from the Moscow strain of ECTV lacks these GAG-binding sites and could not be detected attached to the cell surface ^19^. This suggests that the interaction of the ECTV IL18-BP to the cell surface does not confer an advantage to the virus, since no major differences in virulence have been reported for these two ECTV strains ^20^. Both strains are extremely virulent when tested in the footpad model of infection with susceptible mice, and their median lethal dose has been reported to be under 5 plaque-forming units ^20, 21^.

Another striking and unique feature of vIL-18BP from MCV is the presence of a furin cleavage target site separating the GAG-binding region from the IL-18-binding domain ^11^. Thus, MCV may secrete a GAG-binding vIL-18BP to block IL-18 activity around the site of infection, where virus replication takes place, but also produces a non-GAG-binding form of this inhibitor that could exert its function at more distant sites. This furin cleavage feature could be transferred to other poxviruses to analyze in infection models the impact that blocking IL-18 at different sites will have on pathogenesis.

Since MCV cannot be grown in tissue culture and genomic manipulations are not feasible, evaluation of the contribution of the vIL-18BP to pathogenesis has been carried out in mouse models of infection with VACV ^22, 23^ and ECTV ^12^. Animals infected with viruses lacking vIL-18BP expression suffered a milder illness with reduced weight loss in the case of VACV and decreased levels of virus replication in the case of ECTV compared to wild-type infections. In both cases, these effects were most likely due to increased IFN-γ levels as a consequence of elevated local natural killer cell activation observed after infection in the absence of the vIL-18BP. It is well established that levels of expression of some type I cytokines, including IFN-γ, correlate with resistance to mousepox ^24, 25^. Whereas the relevance of blocking IL-18 during infection has been investigated, the specific contribution of the vIL-18BP GAG-binding properties to poxvirus pathogenesis remains unknown.

## vIFNα/βBP

IFN-I (IFNα and β) is one of the most potent antiviral cytokines of the first line of defense against viral infections ^26^. Among the multiple strategies employed by poxviruses to evade the antiviral effects of IFN-I ^27, 28^, the most straightforward is the secretion of a vIFNα/βBP that sequesters IFN-I prior to its interaction with the specific cellular receptor. This protein was first described in VACV ^29, 30^ and then found to be highly conserved in most virulent poxviruses, such as VARV, ECTV, or MPXV ^25, 31^. The viral protein is composed of three immunoglobulin-like domains, a structure unrelated to host IFN-I receptors, which show fibronectin type III domains. This unique structure may confer the viral protein its high potency as an IFN-I inhibitor and the ability to block IFNs from a variety of species, a property that contrasts with the high species specificity of the cellular counterpart. Deletion of the gene encoding the vIFNα/βBP from VACV and ECTV genomes caused attenuated phenotypes using two different routes of inoculation in mice ^30, 32^. The high attenuation of ECTV (>10 ^6^-fold) showed that the vIFNα/βBP is one of the most potent virulent factors identified so far in poxviruses ^32^. The critical role of the vIFNα/βBP in virulence was confirmed by the ability of antibodies against the vIFNα/βBP, blocking its biological activity, to efficiently protect mice from the highly virulent ECTV ^33^.

Though lacking a transmembrane domain, the vIFNα/βBPs can also interact with GAGs on the surface of infected ( Figure 1) as well as non-infected cells to prevent the IFN-induced antiviral state in cells surrounding the site of infection and to facilitate virus spread ^34, 35^. We identified the GAG-binding sites in vIFNα/βBPs within the first immunoglobulin domain at the amino-terminal region of the protein. By site-directed mutagenesis, we substituted the basic residues within VARV, MPXV, and VACV vIFNα/βBPs to engineer protein versions lacking cell surface-binding ability while leaving their IFN-I-inhibitory activities intact ^35^. It is tempting to speculate that this mutant version of vIFNα/βBP could exert its inhibitory role at more distant tissues than the wild-type version, representing a more potent inhibitor for anti-IFN therapies. However, this possibility remains unexplored.

As stated above, the relevance of the vIFNα/βBPs as virulent factors is well known. The absence of vIFNα/βBP leads to host control of the poxvirus infection: reduced virus spreading and replication in major target organs, absence of clinical signs of illness, and increased survival rates ^32, 34^. The abolishment of the cell-binding properties of the vIFNα/βBP could also drastically affect its efficacy to neutralize the IFN-I host response, particularly in the specific tissues where the virus is replicating, but this possibility has not yet been analyzed.

Given the variety of biological processes in which proteoglycans (GAG-rich signaling receptors) participate ^36^, the binding of viral immunomodulatory proteins to the cell surface raises the possibility of additional and unknown roles for these proteins, such as the triggering of intracellular signaling. In the case of the vIFNα/βBPs, we recently explored this possibility by using an RNAseq-based approach. We could not detect any gene expression changes induced after VACV vIFNα/βBP binding to a mouse cell line ^37^, suggesting that prevention of IFN-I signaling through host IFN-I receptors is the unique function at the cell surface.

Strategies to generate safer and more immunogenic VACV-based vaccines against virulent poxviruses, such as VARV or MPXV, often imply the removal, intentional or not, of diverse immunomodulatory genes from the VACV genome. This is the case for modified vaccinia Ankara (MVA), an attenuated VACV strain lacking, among other proteins, a functional vIFNα/βBP and able to induce a potent and protective immune response ^38^, which has been further optimized through the additional deletion of the vIL-18BP-encoding gene ^39^. MVA could represent an interesting opportunity to evaluate whether attachment to the cell surface may influence safety or immunogenicity (or both) of poxvirus vaccines. The analysis of the immune response elicited by MVA after reinsertion of vIFNα/βBP and vIL-18BP mutant versions, unable to bind to cell surfaces while keeping intact their ligand-binding features, would provide relevant data.

## The A41 and M-T1 vCKBPs

Chemokines are a family of small chemoattractant cytokines released from diverse immune cells that mediate the migration of leukocytes to sites of infection. As highly basic proteins, chemokines interact with GAGs from the endothelium to generate chemotactic gradients and also to facilitate their presentation to specific G-protein-coupled receptors (GPCRs) located at the surface of leukocytes, their target cells ^40^. One of the mechanisms employed by poxviruses to prevent chemokine function is the secretion of viral proteins, unrelated to host receptors and named vCKBPs, that bind chemokines with high affinity and inhibit leukocyte migration ^41– 43^.

Two vCKBPs have been found to interact additionally with GAGs at the cell surface: E163, the A41 orthologue encoded by ECTV, and M-T1 encoded by myxoma virus ^44, 45^. The properties of these vCKBPs illustrate different strategies to modulate chemokine activity ( Figure 1). The A41 family of vCKBPs binds a reduced set of CC and CXC chemokines with high affinity through the GAG-binding domain on the chemokine but does not block the interaction of chemokines with cellular receptors and the subsequent activation of signaling ^44, 46^. This type of interaction would impede the normal binding of the chemokine to GAGs and the correct formation of a chemokine gradient and explain why A41 is not able to inhibit chemokine-induced leukocyte migration *in vitro* while it blocks leukocyte migration to sites of infection in animal models ^47^. By contrast, M-T1 binds to a large set of CC chemokines through their GPCR-binding domain to prevent their interaction with the leukocyte receptor and the induction of cell migration.

The contribution to poxvirus pathogenesis has been examined *in vivo* for both vCKBPs using the corresponding virus deletion mutants. The A41 VACV deletion mutant (deletion of E163 in ECTV has not yet been reported) caused more severe lesions than the wild-type virus in a mouse intradermal model of infection, and this was due to an enhancement of the infiltration of inflammatory cells to the sites of infection that facilitated virus clearance ^47^. Moreover, immunization with VACV MVA lacking A41L confers better protection than control viruses against a virulent VACV strain ^48^. As in the case of A41, myxoma virus infection of European rabbits in the absence of M-T1 increased the number of infiltrating leukocytes to the site of infection during the initial days of infection; however, this increase was not effective enough to produce changes on disease progression or on the overall mortality ^49^.

The GAG-binding properties may provide a retention mechanism for these vCKBPs at the initial sites of infection. Given that two canonical GAG-binding sequences have been described at the carboxy-terminal region of both vCKBPs ^44, 45^, it would be of special interest to analyze the ability of E163/A41 and M-T1 to modulate leukocyte infiltration after mutagenesis of their GAG-binding sites.

When the contribution of vCKBPs to pathogenesis is examined, one additional aspect should be considered: most poxviruses encode diverse vCKBPs, some of them with broad chemokine-binding specificity. This means that the virulence phenotype of a particular poxvirus vCKBP deletion mutant might be masked by the biological action of the remaining vCKBPs.

## The poxviral inhibitor of complement

Once activated, the complement system proteolytic cascade directly acts to destroy virions and lyse infected cells, and this turns out to be critical in the control of many viral infections ^50– 52^. To avoid complement activation where undesirable, host cells express numerous regulatory proteins that bind complement components, such as C3b or C4b, and function as cofactors for serine proteases, accelerating the inactivation of these critical complement components ^53^. Again, to circumvent this part of the innate host response, poxviruses have incorporated a secreted viral protein that binds C3b and C4b and functions in a similar manner to host complement regulatory proteins, promoting complement inactivation ^54^.

This viral inhibitor of the complement system was first described in VACV ^55, 56^ and then, based on amino acid sequence similarity, was found in VARV, CPXV, MPXV, and ECTV ^57– 59^. Although the poxvirus complement control protein is not a cytokine decoy receptor, we have included it in this review because it is also a secreted immunomodulator that binds C3b and C4b in solution and is able to attach to the cell surface. Similar to the host counterparts, the viral complement inhibitor is composed of three or four short consensus repeats. In the case of VACV (named VCP for VACV complement protein) and VARV (named SPICE for smallpox inhibitor of complement enzymes), the attachment to the cell surface was initially described to occur after interaction with GAGs ( Figure 1), mainly through putative GAG-binding sites at short consensus repeats 1 and 4 ^60, 61^. Host complement regulatory proteins also display GAG-binding activity, but the affinity for GAGs is much higher in the case of the poxvirus homologues ^57^. More recently, it was shown that VCP and SPICE are also anchored at the cell surface through an intermolecular disulfide bridge with the viral protein A56 ^62, 63^. It has been speculated that localization at the cell surface enables VCP dimerization, increasing its efficiency to bind C3b and C4b ^62, 63^.

Viruses lacking the complement control protein are attenuated ^64^, and complement-deficient mice (C3 ^−/−^) exhibited increased mortality rates after ECTV infection compared with resistant C57BL/6 mice ^65^, indicating an important contribution of the complement system to poxvirus pathogenesis. Moreover, the corresponding VACV deletion mutant produced smaller lesions than those induced by wild-type virus in an intradermal model of infection ^64^, and the same result was observed after the abolishment of VCP function using monoclonal antibodies prior to infection ^66^. Consistent with these results, less-virulent strains of MPXV encode truncated versions or even lack the complement control proteins ^57, 67^.

A recombinant VACV expressing a mutant version of VCP that cannot locate at the cell surface exhibited a modest attenuation, similar to that observed with the VCP deletion mutant after intranasal infection of mice. In contrast, in the intradermal model of infection, the virulence phenotype of the virus expressing a secreted VCP was intermediate between wild-type and deletion mutant viruses ^62^. Although the attenuation was modest, these results suggest that VCP binding to the cell surface or its dimerization or both contribute to poxvirus pathogenesis. However, it cannot be ruled out that the GAG-binding properties are related to additional immunomodulatory functions of VCP, such as protection from cytotoxic cells ^68^.

## Concluding remarks

Modulation of immune response is crucial for an efficient poxvirus infection, and viruses have optimized their immune evasion mechanisms. The expression of secreted cytokine decoy receptors is an efficient strategy of immune modulation, but experimental evidence indicates that attachment of the secreted decoy receptors to the cell surface might enhance their immunomodulatory activities. Future research should address two related questions. The first is to explore additional poxvirus immune regulatory proteins with GAG-binding properties through novel non-canonical sequences or even GAG-independent mechanisms. For example, the expression of tumor necrosis factor receptor (TNFR) activity was detected at the surface of cells infected with VACV strains USSR, Lister and Evans ^69^, and the viral TNFR CrmE was later found to encode such membrane-bound activity, but the mechanism by which CrmE is anchored at the cell surface was not elucidated ^70^. The second is to discriminate the specific contribution of the GAG-binding properties to poxvirus pathogenesis, combining mutagenesis approaches and appropriate animal models of infection.

A better understanding of the advantage that the cell surface attachment confers to the viral cytokine receptors will provide relevant information to improve the potency of host cytokine decoy receptors. Secreted versions of cytokine receptors are produced naturally to control the immune response and prevent immunopathology, and recombinant versions of soluble TNFRs are used in the clinic to treat a variety of inflammatory conditions such as rheumatoid arthritis, ankylosing spondylitis, or psoriatic arthritis ^71^. A better understanding of the mechanism of inhibition of viral cytokine decoy receptors will be of interest beyond viral pathogenesis, as it may help us to design better anti-inflammatory medicaments.

## Abbreviations

CPXV, cowpox virus; ECTV, ectromelia virus; GAG, glycosaminoglycan; GPCR, G-protein-coupled receptor; IFN-γ, interferon-gamma; IL; interleukin; IL-18BP, interleukin-18-binding protein; MCV, molluscum contagiosum virus; MPXV, monkeypox virus; MVA, modified Ankara virus; SPICE, smallpox inhibitor of complement enzymes; TNFR, tumor necrosis factor receptor; VACV, vaccinia virus; VARV, variola virus; vCKBP, viral chemokine-binding protein; VCP, vaccinia virus complement control protein; vIFNα/βBP, viral interferon type I binding protein; vIL-18BP, viral interleukin-18-binding protein.
